# The Anatectic

**Published:** 1885-08

**Authors:** 


**The Analectic.** A Monthly Periscopic Summary of the Progress of Medical Science. Edited by WALTER S. WELLS, M. D. Volume I. New York and London: G. P. Putnam's Sons, The Knickerbocker Press. 1884.

This is a new venture in medical publications, and is planned to meet "the increasing requirement on the part of the profession for frequent analysis and classification of the immense number of items of practical import to be found scattered throughout the mass of current medical literature." It is well known that the medical literature of the present day has become so voluminous that the active practitioner finds his limited leisure unequal to the requirements. This work aims to furnish a brief abstract of the best thought of the day, and to condense, in a limited space, all the advances made in the various departments of medicine. The reputation of the publishers is an ample guarantee that this plan will be faithfully carried out.

